# An Inverse Method for Measuring Elastoplastic Properties of Metallic Materials Using Bayesian Model and Residual Imprint from Spherical Indentation

**DOI:** 10.3390/ma14237105

**Published:** 2021-11-23

**Authors:** Mingzhi Wang, Weidong Wang

**Affiliations:** 1 School of Mechano-Electronic Engineering, Xidian University, Xi’an 710071, China; 2 School of Mechanical Engineering, Northwestern Polytechnical University, Xi’an 710072, China

**Keywords:** indentation experiment, metallic materials, elastoplastic properties, mechanical measurement, Bayesian model, inverse problem

## Abstract

In this paper, an inverse method is proposed for measuring the elastoplastic properties of metallic materials using a spherical indentation experiment. In the new method, the elastoplastic parameters are correlated with sub-space coordinates of indentation imprints using proper orthogonal decomposition (POD), and inverse identification of material properties is solved using a statistical Bayesian framework. The advantage of the method is that model parameters in the numerical optimization process are treated as the stochastic variables, and potential uncertainties can be considered. The posterior results obtained from the measuring method can provide valuable probabilistic information of the estimated elastoplastic properties. The proposed method is verified by the application on 2099-T83 Al-Li alloys. Results indicate that posterior distribution of material parameters exhibits more than one peak region when indentation load is not large enough. In addition, using the weighting imprints under different loads can facilitate the uniqueness in identification of elastoplastic parameters. The influence of the weighting coefficient on posterior identification results is analyzed. The elastoplastic properties identified by indentation and tensile experiment show good agreement. Results indicate that the established measuring method is effective and reliable.

## 1. Introduction

The indentation test has long been used as an efficient and versatile way to measure the basic mechanical parameters of materials, e.g., hardness and yield strength [1,2,3]. The rapid development of high-resolution load/depth sensing technologies has inspired a tremendous interest in measuring the strain hardening properties of various materials by indentation experiments [4,5]. The main advantage of the indentation test is that the experiment process is very simple and versatile [5,6,7]. It can also be used for the in situ measurement of material properties in a very local region, where the conventional uniaxial experiment is not applicable [5,6,7].

The multiaxial and complex stress states of materials under a spherical indenter are very different from those experienced in a uniaxial test. To obtain meaningful measuring results, e.g., mechanical properties, by indentation, researchers have resorted to the finite element (FE) simulation and inverse analysis. The use of the FE method makes it possible to build potential correlations between material constitutive parameters and the mechanical responses obtained from the indentation test. Based on this, considerable efforts have been made and many promising methods [5,6,7,8,9,10,11,12] have been established to obtain the elastoplastic properties of material by indentation experiments. Among these previous works [5,6,7,8,9,10,11,12,13,14], the indentation load-displacement curve has always been considered, and iterative optimization algorithms have been adopted to minimize the discrepancy between experiment and simulations [5,6,7,8,9,10,11,12,13,14]. Numerical methods for measuring the elastoplastic properties of materials are essentially based on the deterministic optimization process, with extensive FE simulations using parameter iterative updating, e.g., the gradient-based optimization [6,11], and the Nelder–Mead search method [13,14]. These optimization methods were well developed, and some experiment and algorithm convergence issues have been well described [6,11,13,14]. However, the material constitutive parameters in these previous studies were regarded as the deterministic values, and potential uncertainties in the model and experiment were not considered [15,16,17].

It is noted that the measuring data observed from the indentation experiment can be easily influenced by many uncertain factors, e.g., surface roughness and material heterogeneity [15,18,19], and usually exhibit disturbance [20]. The iteration trajectory of the optimization process may be influenced by the disturbance of experiment error and easily be trapped in the local minimum [20]. Therefore, it is very necessary to consider the uncertainties in measuring the material parameters using indentation experiments and numerical algorithms. Moreover, in many indentation studies [5,6,7,8,9,10,11,12], the load-displacement curve was mainly considered in numerical modeling in the parameter identification process. However, obtaining a unique solution is a challenge when only the indentation load-displacement curve is considered in the parameter identification process [2,9,11,12]. To alleviate this problem, some researchers [6,9,11] introduced the pile-up value as the extra information by weighting the load-displacement curve and the pile-up value. However, the above two physical amounts are essentially different, and their numerical magnitudes are usually not compatible. The highly nonlinear shape of the residual indentation imprint makes it very hard to determine a correct pile-up value.

Therefore, using the whole indentation imprint in the parameter identification process is preferred [21,22]. The spherical indentation profile has been proved to be more sensitive to the plastic parameters of materials than the load-displacement curve [13]. In the work by Clyne et al. [23], the advantages of using the indentation imprint were summarized. It does not require the extra indentation loading–unloading data, thus eliminating the uncertainties involved in machine compliance. In addition, the use of the whole residual imprint can effectively circumvent the need for accurately measuring the pile-up value. The whole indentation imprint has been used in determining the residual stresses of materials [24,25], strain hardening properties of metals [6,21,22], and anisotropic properties of material [26,27]. In particular, the whole indentation imprint was regarded as the “fingerprint,” to reveal the orientation-dependent deformation mechanisms of single crystals [28,29,30].

In present work, an inverse method is established for the measurement of material elastoplastic properties by spherical indentation imprint. The whole indentation experiment imprint will be used as the experiment information in the numerical computation. The inverse problem is solved using a statistical Bayesian inference model. The elastoplastic properties and coordinates of indentation imprint are well correlated in the sub-space. The posterior sampling result provides meaningful probabilistic information of the inverse measured elastoplastic properties, and it helps to further interrogate the uniqueness of the parameter identification. The probabilistic distribution of elastoplastic properties identified by the measuring method are very useful in terms of the materials selection, plasticity modeling, and structure design [31,32,33]. Material, tensile, and indentation experiments are presented in Section 2. The mathematical methods, FE simulation, and procedures of the measuring method are described in Section 3. Results and discussion are described in Section 4, and conclusion is summarized in Section 5.

## 2. Material and Experiments

### 2.1. Material

**Figure 1:**
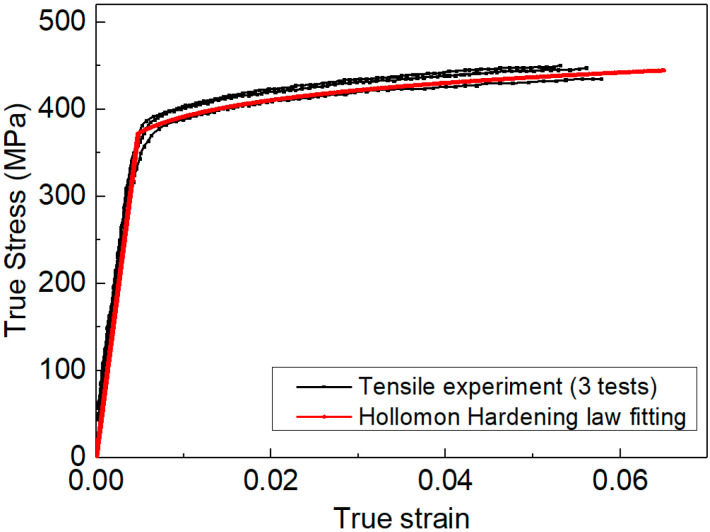
The stress–strain curves obtained from the tensile experiment.

The materials studied here were 2099-T83 Al-Li alloys. Because of the excellent physical performance, e.g., low density/high strength, this material has been widely used in the aerospace manufacturing industry [34,35]. The tensile experiment adhering to the ASTM standard was used to obtain the tensile properties of this material, so that the elastoplastic measured by indentation could be comparable. Figure 1 shows the stress–strain curve obtained from the tensile experiment. In the study, strain hardening of this material was described by the Hollomon constitutive law, as shown in Figure 1. The fitting parameters are listed in Table 1.

The Hollomon hardening law can be used to describe the tensile behaviors of most metallic alloys, and it is described in Equation (1) [2,6].

(1) $$\sigma =E\varepsilon ,\;\sigma \leq \sigma_{y}\;\mathrm{and}\;\sigma =E^{n}\sigma_{y}^{1-n}\varepsilon^{n},\;\sigma \geq \sigma_{y}$$

where *E* is the elastic modulus, n is the strain hardening exponent, and $\sigma_{y}$ is yield stress. In Figure 1, the Hollomon law provides a very accurate description of the strain hardening behaviors of 2099-T83 Al-Li alloys.

### 2.2. Indentation Experiment

**Figure 2:**
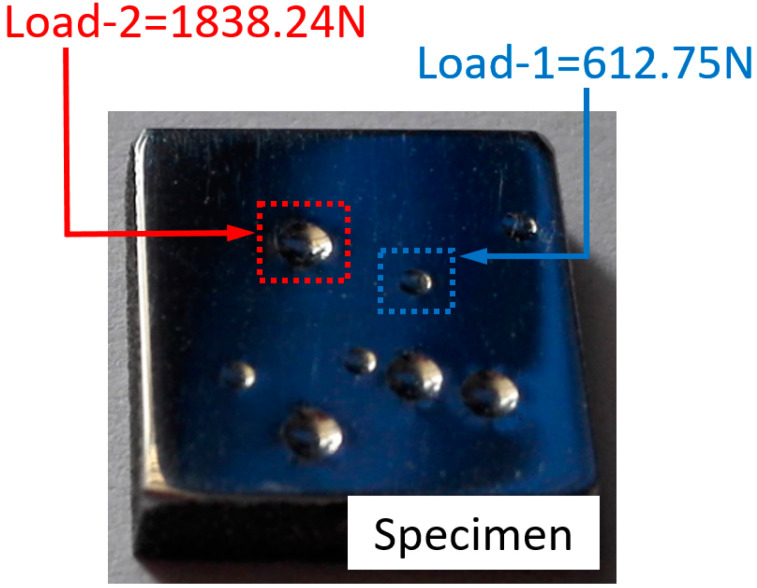
Cubic specimen and residual indentation imprint under two different indentation loads.

The spherical indentation experiment was performed using a Brinell Hardness tester. The radius of the indenter was 1.25 mm, and it was made of a tungsten carbide ball. The cubic specimen with length 10 mm, width 10 mm, and height 3 mm was prepared for indentation test. The surface of the specimen was carefully polished to a mirror finish, so that the influence of surface roughness was negligible. An indentation test was performed at room temperature, and the load was applied on the indenter to press against the surface of specimen up to its maximum value. Holding time was 15 s, and then the indenter was unloaded. Figure 2 shows the specimen and the indentation imprint under two different loads.

**Figure 3:**
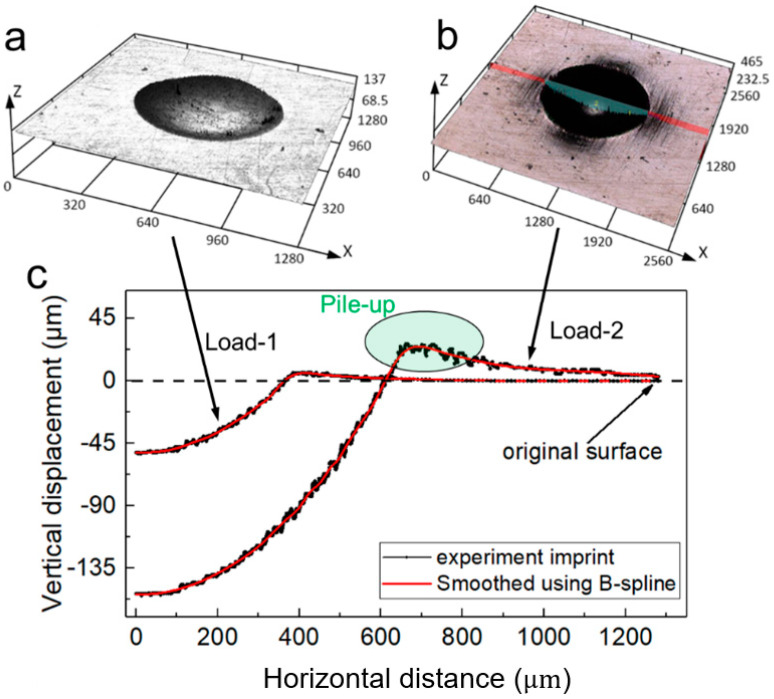
The measured indentation imprints using a laser confocal microscope: (**a**) imprint mapping under indentation Load-1; (**b**) imprint mapping under indentation Load-2; (**c**) corresponding 2D imprint snapshots (unit: µm).

Here, Figure 2 clearly shows the imprints under different indentation loads. The indentation imprint was measured by a 3D laser confocal microscope (OSL4000), and measuring results are shown in Figure 3. In Figure 3a,b, the 3D imprints were obtained from two different loads, Load-1: 612.75 N and Load-2: 1838.24 N, respectively. The corresponding 2D imprint snapshots are shown in Figure 3c. In the experiment process, the distance between two adjacent imprints should be large enough to avoid the potential influence of residual stress on the shape of imprint under the next indentation.

Here, the B-spline curve was used to smooth and interpolate the measuring data, in order to eliminate the potential noise. In Figure 3c, the imprint snapshots exhibit obvious pile-up behavior, and this is especially obvious for the imprint snapshot under a higher indentation load, e.g., Load-2: 1838.24 N. The indentation scale in the study was macroscale, as shown in Figure 3, and the reported average grain size of 2099-T83 was about 7.6 μm [35]. Thus, the influence of grain size and anisotropy of a single grain on the whole indentation imprint was negligible.

It was noted that the size/magnitude of the indentation imprints obtained from two loads were very different. Moreover, the imprint snapshot obtained from a larger indentation load exhibited a higher pile-up behavior. Thus, the parameter identification results obtained from different indentation loads may be different. In the study, those two different indentation imprints were separately considered in the numerical method, and the posterior identification results were compared and analyzed.

## 3. The Basic Methods

### 3.1. Mathematical Analysis Tools

#### 3.1.1. Correlate Elastoplastic Parameters with Indentation Imprint Snapshots Using a POD-Based Numerical Model

The vertical displacement values of the imprint snapshot are saved in vector $S_{i}^{j}$. Here, vector $S_{i}^{j}$ is used as parametric representation of the shape of an imprint snapshot under the ith prescribed indentation load. Thus, $S_{i}^{j}\in R^{N}$, R represents the set of real numbers, and N is the total number of the node values in imprint vector $S_{i}^{j}$. Here, the superscript j in vector $S_{i}^{j}$ represents the jth imprint snapshot in imprint matrix $S_{i}$, that will correspond to the jth combination in the elastoplastic parameter design space. To correlate the coordinate of indentation imprint in sub-space with elastoplastic properties using the POD algorithm, the imprint snapshot database $O_{s}$ should be firstly established using extensive FE simulations. In the study, the imprint snapshots under two indentation loads, Load-1 and Load-2, were considered, and thus the imprint database $O_{s}$ can be mathematically expressed in Equation (2).

(2) $$O_{s}=\left[\begin{array}{c} S_{1}\\ S_{2} \end{array}\right]={\left[\begin{array}{c} \begin{array}{cc} \begin{array}{cc} S_{1}^{1} & S_{1}^{2} \end{array} & \begin{array}{cc} \cdots & S_{1}^{M} \end{array} \end{array}\\ \begin{array}{cc} \begin{array}{cc} S_{2}^{1} & S_{2}^{2} \end{array} & \begin{array}{cc} \cdots & S_{2}^{M} \end{array} \end{array} \end{array}\right]}_{2\times M}$$

where $O_{s}$ is the imprint database built by extensive simulations using the FE model, with respect to M combinations from the elastoplastic parameter design space. In the database $O_{s}$, $S_{i}$ represents the imprint matrix obtained from the ith indentation load, and each column $S_{i}^{j}$ in matrix $S_{i}$ represents the imprint snapshot corresponding to the jth combination of elastoplastic parameters.

Based on the definition in Equation (2), the deviation matrix can be expressed as

(3) $$O_{s}^{dev}=\left[\begin{array}{c} S_{1}^{dev}\\ S_{2}^{dev} \end{array}\right]=\left[\begin{array}{c} \begin{array}{cc} \begin{array}{cc} S_{1}^{1}-{\bar{S}}_{1} & S_{1}^{2}- \end{array}{\bar{S}}_{1} & \begin{array}{cc} \ldots & S_{1}^{M}-{\bar{S}}_{1} \end{array} \end{array}\\ \begin{array}{cc} \begin{array}{cc} S_{2}^{1}-{\bar{S}}_{1} & S_{2}^{2}- \end{array}{\bar{S}}_{1} & \begin{array}{cc} \ldots & S_{2}^{M}-{\bar{S}}_{1} \end{array} \end{array} \end{array}\right]$$

where $S_{i}^{dev}$ is the covariance matrix of the imprint matrix $S_{i}$. In Equation (3), ${\bar{S}}_{i}$ represents the averaged imprint snapshot of matrix $S_{i}$, and it is defined as: ${\bar{S}}_{i}=\left(1/M\right)\sum_{j=1}^{M}S_{i}^{j}$.

In the study, the POD algorithm is used to generate the sub-space coordinates of indentation imprints, and each indentation imprint in matrix $S_{i}$ can be linearly represented by the orthogonal basis using the sub-space coordinates, as expressed in Equation (4).

(4) $$S_{i}^{j}={\bar{S}}_{i}+U_{i}\alpha_{i}^{j}={\bar{S}}_{i}+\sum_{k}^{N}\alpha_{i}^{jk}U_{i}^{j},\;\mathrm{with}\;i=1,\;2$$

where $U_{i}$ is the basis matrix, and each vector $U_{i}^{j}$ in matrix $U_{i}$ is the orthogonal basis. $\alpha_{i}$ represents the sub-space coordinate matrix of $S_{i}$. $\alpha_{i}$ = [$\alpha_{i}^{1}$,$\alpha_{i}^{2}$,…,$\alpha_{i}^{M}$], and the kth value in $\alpha_{i}^{j}$ is defined as $\alpha_{i}^{jk}$. So, $\alpha_{i}^{j}$ is the sub-space coordinate of the corresponding imprint snapshot $S_{i}^{j}$.

In the POD algorithm, the orthogonal basis in $U_{i}$ is obtained from the Singular Value Decomposition (SVD) of matrix $S_{i}^{dev}$, as expressed in Equation (5).

(5) $$S_{i}^{dev}=U_{i}S_{i}V_{i}^{T},\;\mathrm{with}\;i=1,\;2\;$$

where $U_{i}$ is the orthogonal basis, and it is defined as $U_{i}$ = [$U_{i}^{1}$,$U_{i}^{2}$,…,$U_{i}^{N}$]. Here, the subscript i indicates the situation is under the ith indentation load. $V_{i}$ is a unitary matrix, with $V_{i}V_{i}^{T}=I$. $S_{i}$ is the diagonal matrix, and the corresponding eigenvalues are included in this matrix.

Therefore, the coordinate $\alpha_{i}$ of indentation imprint in matrix $S_{i}$ can be obtained by using Equation (6).

(6) $$\alpha_{i}={\left(U_{i}^{T}U_{i}\right)}^{-}\;U_{i}^{T}\left(S_{i}-{\bar{S}}_{i}\right),\;\mathrm{with}\;i=1,\;2$$

where ${\left(U_{i}^{T}U_{i}\right)}^{-}$ represents the inverse matrix of $U_{i}^{T}U_{i}$. In the study, all modes in basis matrix $U_{i}$ will be considered, so that very high numerical accuracy in Equation (4) can be guaranteed [36,37].

The POD algorithm is a very efficient protocol for capturing the principal deformation features of indentation imprint snapshots [36,37,38]. In the study, it provided the optimal representation of an imprint snapshot using the orthogonal basis in well-established sub-space, e.g., the expression in Equation (4). The correlation between coordinate $\alpha_{i}$ of indentation imprints in sub-space and elastoplastic parameters c are established using the relationship described in Equation (7).

(7) $$\beta_{i}^{j}\left(c_{j}\right)=k^{T}\left(c_{j}\right)a_{i}^{j},\;\mathrm{with}\;i=1,\;2$$

where $\beta_{i}$ is the transposition of sub-space coordinate matrix $\alpha_{i}$, and each row in matrix $\beta_{i}$ is defined as $\beta_{i}^{j}$. $k^{T}\left(c_{j}\right)$ is the polynomial basis matrix, and $a_{i}^{j}$ is the regression vector that is used to parameterize the relation between vector c and each column in $\beta_{i}$. Here, the regression coefficients matrix is $a_{i}$, as $a_{i}$ = [$a_{i}^{1}$,$\;a_{i}^{2}$,…,$\;a_{i}^{M}$]. c is a vector that includes the combination of elastoplastic properties in the parameter design space with $c\in R^{l}$, and l is dimension of c. Thus, Equation (7) essentially represents the direct parametric relations between elastoplastic parameters c and coordinate $a_{i}$ in the sub-space. The details on how to approximate the parametric relationship in Equation (7) will be given in Appendix A.

#### 3.1.2. Bayesian Inference in Sub-Space of Indentation Imprints

The statistical Bayesian inference is a very effective inverse computation protocol, and it has received a great attention in identification of model parameters, by using the information measured from actual experiments [15,32,33]. The Bayesian inference formula is described in Equation (8) [15,32,33]

(8) $$f\left(c|S_{\mathrm{eff}}^{\exp},\Phi_{c}\right)=\frac{f\left(S_{\mathrm{eff}}^{\exp}|c,\Phi_{c}\right)\cdot f\left(c|\Phi_{c}\right)}{f\left(S_{\mathrm{eff}}^{\exp}|\Phi_{c}\right)}$$

where $\Phi_{c}$ is the constitutive model that describes the hardening behavior of materials under indentation, e.g., the Hollomon hardening law. Vector c includes the elastoplastic properties that must be identified according to the information measured from the indentation experiment. $S_{\mathrm{eff}}^{\exp}$ is a vector, and it represents the effective measuring amount obtained from the indentation experiment. In Equation (8), $f\left(c|\Phi_{c}\right)$ is the prior information of material elastoplastic parameters in c. $f\left(S_{\mathrm{eff}}^{\exp}|\Phi_{c}\right)$ is an evidence/constant term that is not dependent on the variables in c [15,16]. $f\left(c|S_{\mathrm{eff}}^{\exp},\Phi_{c}\right)$ is the posterior distribution function (PDF) of unknown elastoplastic parameters in c. $f\left(S_{\mathrm{eff}}^{\exp}|c,\Phi_{c}\right)$ is the likelihood function, which represents how likely the prediction matches the measured experiment amounts under the given hardening law $\Phi_{c}$ and elastoplastic parameters in c [15,16]. In the study, prior information of elastoplastic parameters usually obeyed uniform distribution [15].

The posterior distribution $f\left(c|S_{\mathrm{eff}}^{\exp},\Phi_{c}\right)$ is proportional to the likelihood function, as expressed in Equation (9).

(9) $$f\left(c|S_{\mathrm{eff}}^{\exp},\Phi_{c}\right)\propto f\left(S_{\mathrm{eff}}^{\exp}|c,\Phi_{c}\right)\cdot f\left(c|\Phi_{c}\right).$$

The purpose of the Bayesian inference formula defined in Equation (9) is to infer the unknown elastoplastic properties in c by simply sampling from $f\left(c|S_{\mathrm{eff}}^{\exp},\Phi_{c}\right)$ [15,32]. In Equation (9), prior information $f\left(c|\Phi_{c}\right)$ obeys uniform distribution, and thus the sampling process can be easily implemented on the likelihood function [15,16]. The likelihood function is expressed in Equation (10).

(10) $$f\left(S_{\mathrm{eff}}^{\exp}|c,\Phi_{c}\right)={\left(2\pi \sigma^{2}\right)}^{-\frac{N}{2}}\exp\left\{-\frac{\sum_{j=1}^{N}{\left(S_{\mathrm{eff}\_j}^{\exp}-f_{j}\left(S_{\mathrm{eff}\_j}|c,\Phi_{c}\right)\right)}^{2}}{2\sigma^{2}}\right\}$$

where the term $\sum_{j=1}^{N}{\left(S_{\mathrm{eff}\_j}^{\exp}-f_{j}\left(S_{\mathrm{eff}\_j}|c,\Phi_{c}\right)\right)}^{2}$ represents the error norm, and it can be written as $\|S_{\mathrm{eff}}^{\exp}-f\left(S_{\mathrm{eff}}|c,\Phi \right){\|}^{2}$. Here, $f\left(S_{\mathrm{eff}}|c,\Phi_{c}\right)$ is the predicted amount of $S_{\mathrm{eff}}^{\exp}$, according to the given hardening law $\Phi_{c}$ and elastoplastic parameters in c. $S_{\mathrm{eff}\_j}^{\exp}$ is the jth value in vector $S_{\mathrm{eff}}^{\exp}$, and $S_{\mathrm{eff}\_j}$ is the jth value in vector $S_{\mathrm{eff}}$. The symbol ‖·‖ represent the 2-normal. In Equation (10), $\sigma^{2}$ is the variance, and it is determined using the maximum likelihood estimation (MLE) [15,16,32], as

(11) $$\sigma^{2}=\frac{1}{N}\sum_{j=1}^{N}{\left(S_{\mathrm{eff}\_j}^{\exp}-f_{j}\left(S_{\mathrm{eff}\_j}|c,\Phi_{c}\right)\right)}^{2}=\frac{1}{N}\|S_{\mathrm{eff}}^{\exp}-f\left(S_{\mathrm{eff}}|c,\Phi \right){\|}^{2}.$$

Therefore, the likelihood function is further expressed as

(12) $$f\left(S_{\mathrm{eff}}^{\exp}|c,\Phi_{c}\right)={\left(\frac{N}{2\pi \|S_{\mathrm{eff}}^{\exp}-f{\left(S_{\mathrm{eff}}|c,\Phi_{c}\right)\|}^{2}}\right)}^{\frac{N}{2}}\exp\left(-\frac{N}{2}\right).$$

#### 3.1.3. The Proper Weighting in the Sub-Space of Indentation Imprint Snapshots

In the study, a weighting coefficient λ was introduced in the established measuring method to account for the two imprint snapshots under different indentation loads, e.g., Load-1 and Load-2. Here, the basic assumption was that, weighting of imprint snapshots under different indentation loads can introduce more experiment information in model space, and it helps to facilitate uniqueness in identification of elastoplastic properties. This problem will be discussed in Section 4.3. The weighting of indentation imprints under different indentation loads is expressed as

(13) $$S_{\mathrm{eff}}=\left(1-\lambda \right)S_{1}+\lambda S_{2}$$

where $S_{1}$ and $S_{2}$ are the imprint snapshots obtained from indentation Load-1 and Load-2, respectively. λ is a weighting coefficient, and it is used to weight the source of errors from indentation imprints, $S_{1}$ and $S_{2}$ in the numerical computation.

By using Equation (4), the term $S_{\mathrm{eff}}^{\exp}-f\left(S_{\mathrm{eff}}|c,\Phi_{c}\right)$ in Equation (12) can be expressed using the sub-space coordinates of indentation imprints, and it is expressed as

(14) $$S_{\mathrm{eff}}^{\exp}-f\left(S_{\mathrm{eff}}|c,\Phi_{c}\right)=\left(1-\lambda \right)U_{1}\left[\alpha_{1}^{\exp}-\alpha_{1}\left(S_{1}|c,\Phi_{c}\right)\right]+\lambda U_{2}\left[\alpha_{2}^{\exp}-\alpha_{2}\left(S_{2}|c,\Phi_{c}\right)\right]$$

where $\alpha_{1}^{\exp}$ and $\alpha_{2}^{\exp}$ are coordinates of experiment imprints $S_{1}^{\exp}$ and $S_{2}^{\exp}$ in the sub-space. $\alpha_{1}\left(S_{1}|c,\Phi_{c}\right)$ and $\alpha_{2}\left(S_{2}|c,\Phi_{c}\right)$ are coordinates of the predicted indentation imprints $f\left(S_{1}|c,\Phi_{c}\right)$ and $f\left(S_{2}|c,\Phi_{c}\right)$ in the sub-space.

Therefore, the likelihood function in Equation (12) is expressed as

(15) $$f\left(S_{\mathrm{eff}}^{\exp}|c,\Phi_{c}\right)={\left(\frac{N}{2\pi \|\left(1-\lambda \right)U_{1}\left[\alpha_{1}^{\exp}-\alpha_{1}\left(S_{1}|c,\Phi_{c}\right)\right]+\lambda U_{2}\left[\alpha_{2}^{\exp}-\alpha_{2}\left(S_{2}|c,\Phi_{c}\right)\right]{\|}^{2}}\right)}^{\frac{N}{2}}\exp\left(-\frac{N}{2}\right).$$

In the study, the relation between elastoplastic properties in c and the coordinate matrix $\alpha_{i}$ sub-space was well correlated in Equation (7). Once the imprint database $O_{s}$ was established, the orthogonal basis matrices $U_{1}$ and $U_{2}$ were known based on the established POD model. The sub-space coordinate of experiment imprint snapshots were obtained by using the relations: $\alpha_{1}^{\exp}$ = ${\left(U_{1}^{T}U_{1}\right)}^{-}U_{1}^{T}\left(S_{1}^{\exp}-{\bar{S}}_{1}\right)$ and $\alpha_{2}^{\exp}\;$=$\;{\left(U_{2}^{T}U_{2}\right)}^{-}U_{2}^{T}\left(S_{2}^{\exp}-{\bar{S}}_{2}\right)$, according to the definition in Equation (6). Therefore, the model evaluation of $\alpha_{1}\left(S_{1}|c,\Phi_{c}\right)$ and $\alpha_{2}\left(S_{2}|c,\Phi_{c}\right)$ were readily obtained, and thus, posterior distribution of unknown elastoplastic properties was directly calculated by using Monte Carlo (MC) sampling [32,39] on Equation (15). In the study, the Transition Markov Chain Monte Carlo (TMCMC) [32,33] was used to sample from the PDF in Equation (15). This algorithm can generate a very robust Markov chain, and it will converge to the target posterior PDF. In parameter identification using the indentation test, the inverse problem may sometimes be ill-posed and give the local optimal values. The TMCMC algorithm has been used in many engineering fields [15,39] and it can be very effective, especially for the sampling problem with peak regions and high dimensionality [32,39]. More information about the TMCMC algorithm used here can be found in Refs [32,39].

### 3.2. Finite Element Simulation of Spherical Indentation Test

**Figure 4:**
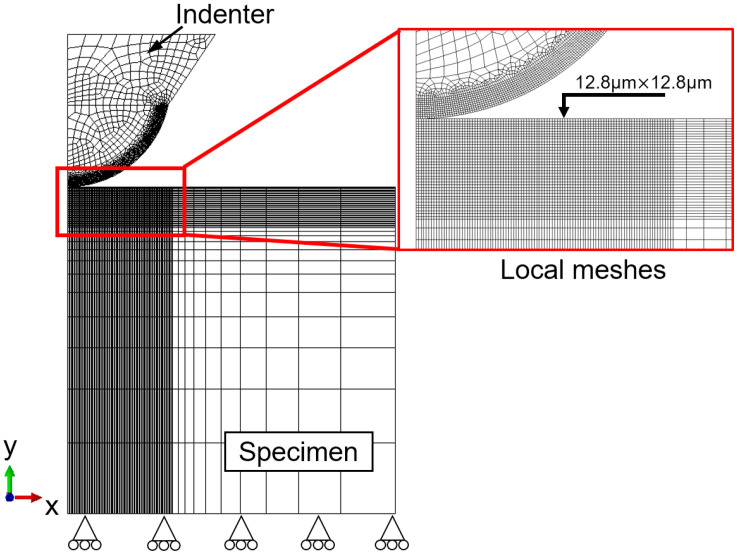
FE simulation model and boundary conditions.

The FE simulation of spherical indentation was implemented using Abaqus/Standard software [40]. Figure 4 shows the established FE model of spherical indentation. The axial–symmetric boundary conditions were considered to account for the axial–symmetric properties for both indentation geometry and material properties, as shown in Figure 4. The height and radius of the specimen were 4 mm, and the radius of the indenter was 1.25 mm. The indenter was regarded as a deformable body, with elastic modulus 600 GPa and Poisson’s ratio 0.23 [41]. Both the specimen and indenter were modeled using CAX4R element type. In the local contact regions between indenter and specimen, very refined meshes were created to improve the accuracy of the FE simulation results. The minimum element size used in the FE model was 12.8 μm. In the FE model, totals of 14,060 and 3693 elements were created, respectively, for the specimen and indenter. The contact friction factor between the surfaces of specimen and indenter was fixed at 0.1, and this is a reasonable value for the contact behavior between metals and a hard indenter [41,42]. The Poisson’s ratio was 0.3 for the specimen [43]. The bottom of the specimen was fixed, and the indenter was pressed against the surface of the specimen up to a maximum load value $P_{\max}$. The indenter was then unloaded gradually.

### 3.3. The Procedures for Identification of Elastoplastic Parameters Using the Established Measuring Method

**Figure 5:**
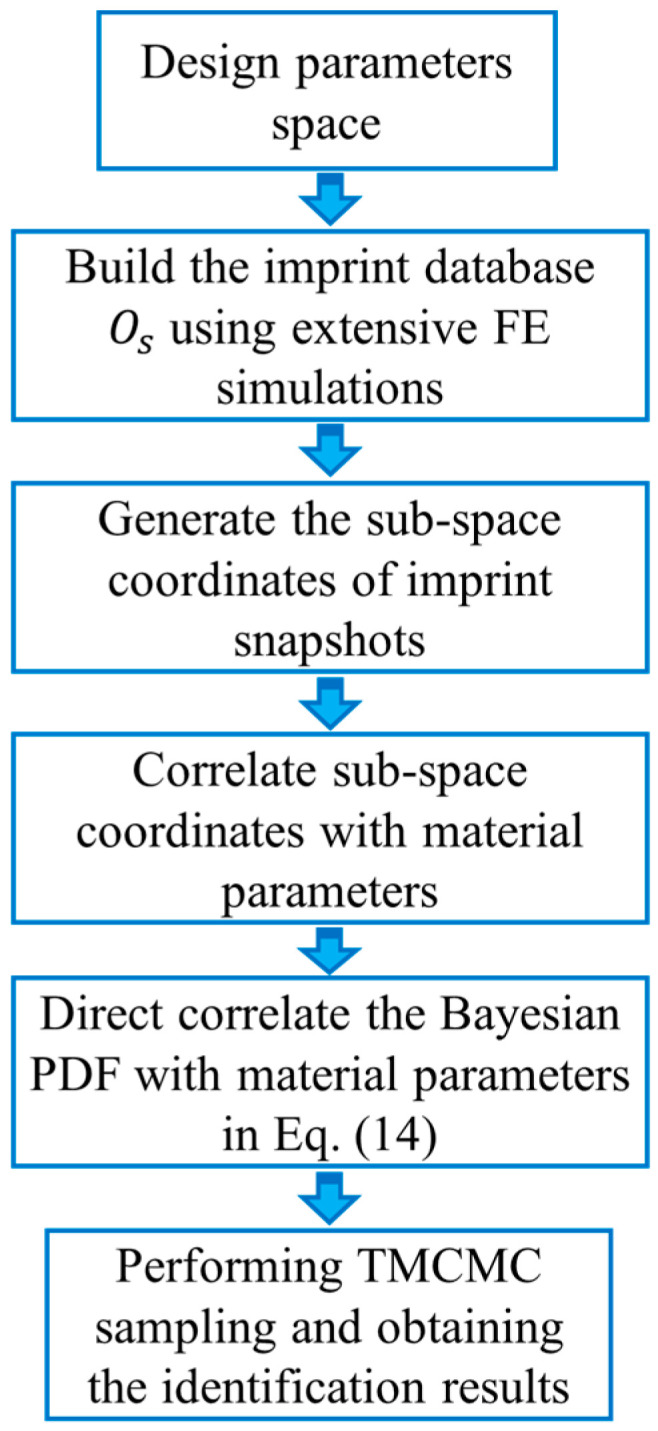
The basic procedures for measuring elastoplastic properties of materials using the established approach.

In this section, detailed procedures on how to obtain the elastoplastic properties using the established measuring method will be described. The elastic and plastic parameters, E, $\sigma_{y}$ and n of the Hollomon law will be inferenced by the established new method and the residual imprint obtained from indentation experiment. Thus, the dimension of vector c is 3. Figure 5 shows the flow diagram of three steps for measuring the elastoplastic parameters using the established measuring method. It was noted that the elastic modulus is also considered in the modeling process of the proposed numerical method. Although the stiffness of the materials can be known beforehand, it actually depends on the base metal type. The level of precision required is relatively low. If the prior known elastic modulus is not very accurate and it has a relatively large discrepancy with respect to the actual elastic value, its use may introduce extra errors, thus greatly reducing the accuracy of the estimated plastic properties, e.g., yield strength and strain hardening exponent. Moreover, it is necessary to determine very accurately the elastic modulus and plastic stress–strain curve simultaneously, for example, in predicting and controlling the springback of sheet metals in plastic forming. The plastic stress–strain curve will influence the plastic deformation of sheet metals greatly, and at the same time the elastic modulus is also a key parameter in predicting the springback of the products.

In the study, three basic steps were needed for establishing the proposed Bayesian inference method, and they are generally described as follows: (1) We designed the elastoplastic parameter space and built the imprint database $O_{s}$ with the assistance of the FE simulations. In the study, the elastoplastic parameters were within: 30 GPa≤E≤110 GPa, $280\;\mathrm{MPa}\leq \sigma_{y}\leq 460\;\mathrm{MPa},$ and 0.005≤n≤0.125, with the intervals ΔE = 20 GPa, $\Delta \sigma_{y}$ = 30 MPa, and Δn = 0.02. Therefore, in the design space, there was a total of 245 combinations of elastoplastic parameter, and M value in Equation (2) was 245. (2) The POD algorithm was performed on the imprint database $O_{s}$, and sub-space coordinates of imprint snapshots were generated. Then, the parametric relationship in Equation (7) was established. (3) We built the direct correlation between elastoplastic parameters and the posterior distribution functions in Equation (15). By performing TMCMC sampling directly on the PDF in Equation (15), the posterior distribution of unknown elastoplastic properties, E, $\sigma_{y}$ and n were obtained. In the study, the total number of samples used in the TMCMC sampling process was 1 $\times \;10^{4}$, and this value was enough to give very stable posterior results in the TMCMC sampling process.

## 4. Results and Discussion

### 4.1. The Measurement of Elastoplastic Parameters Using the Imprint Snapshot under Different Indentation Load/Depth Values

The established measuring method in Section 3 was used to measure elastoplastic properties, E, $\sigma_{y}$ and n of the studied material, based on the imprint mapping obtained from the indentation experiment. Here, the imprint snapshots obtained from two different indentation load values, Load-1 (612.75 N) and Load-2 (1838.24 N), were separately used in the numerical computation. So, two totally different situations were considered in the study. The numerical results obtained from the above situations were compared and analyzed.

**Figure 6:**
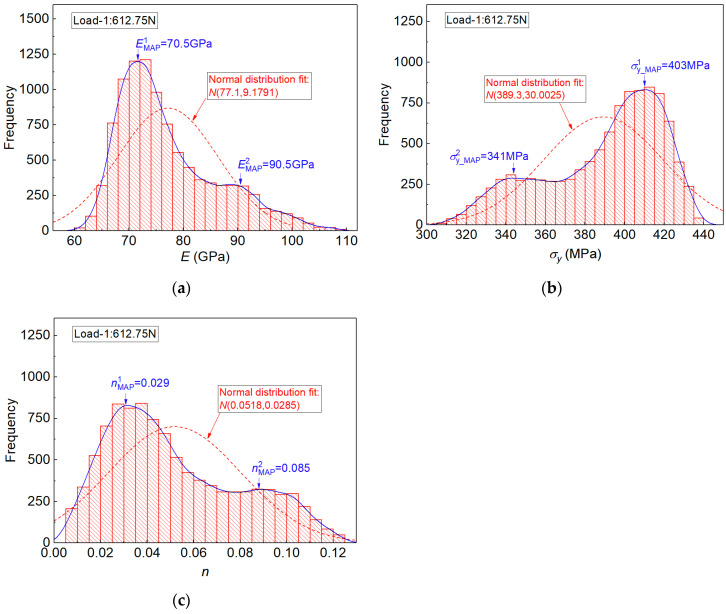
Posterior distribution results of the estimated elastoplastic properties from the established measuring method and experiment imprint snapshots under indentation Load-1: in (**a**) for E, in (**b**) for $\sigma_{y}$ and in (**c**) for n.

**Figure 7:**
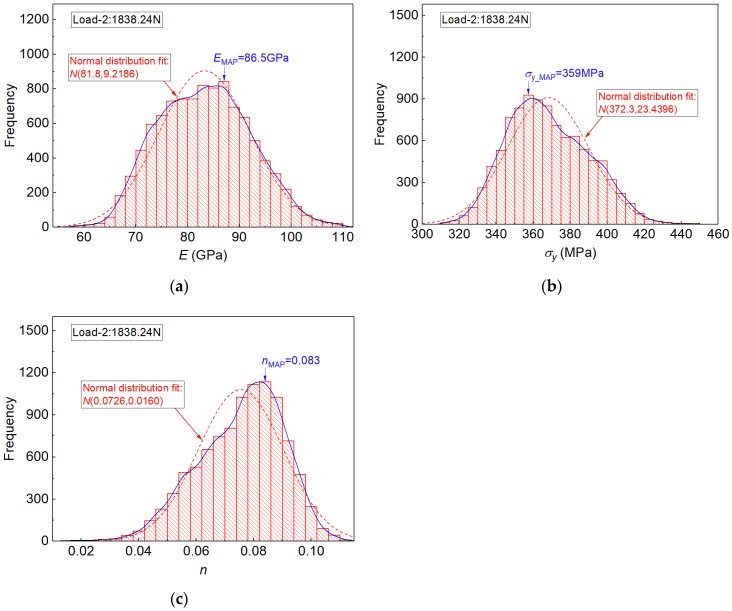
Posterior distribution results of the estimated elastoplastic parameters by the established measuring method and experiment imprint snapshots under indentation Load-2: in (**a**) for E, in (**b**) for $\sigma_{y}$ and in (**c**) for n.

In situation one, only the imprint snapshot under indentation Load-1 was used, and the weighting coefficient λ in Equation (15) was fixed at 0. In situation two, only the imprint snapshot under indentation Load-2 was used, and the weighting coefficient in this situation was fixed at 1. In Equation (15), the coefficient λ was essentially adopted to weight the sources of errors from indentation imprints under two different loads. The influence of coefficient λ on the posterior sampling results will be systematically analyzed in Section 4.3. Figure 6 and Figure 7 show the posterior marginal distributions of the identified elastoplastic properties E, $\sigma_{y}$ and n by the proposed measuring method and single experiment imprint under two different loads (612.75 N and 1838.24 N), respectively, in Figure 6a–c for indentation Load-1, and in Figure 7a–c for indentation Load-2.

In Figure 6, posterior distribution results of material properties obtained from these two indentation loads are very different. In Figure 6a–c, the posterior marginal distribution of each material parameter exhibits more than one peak region, and this is observed from the kernel smoothed curves (blue curves in Figure 6). In situation one, two different maximum posterior (MAP) estimation points were obtained, and they are denoted as MAP point-1 and MAP point-2, as shown in Figure 6a–c. Results indicated the inverse identified elastoplastic parameters E, $\sigma_{y}$ and n were not unique when the experiment imprint under indentation Load-1: 612.75N was used in the sampling process. We noted that the posterior sampling process was realized by using the TMCMC algorithm according to the defined probabilistic function in Equation (15). So the frequency of the TMCMC samples was used as the indicator to reflect the identification results of material parameters, as shown in Figure 6 and Figure 7. The values corresponding to the maximum frequency were regarded as the maximum posterior (MAP) estimation point.

In the study, posterior marginal distributions of elastoplastic properties were fitted using *Normal distribution*, e.g., the dotted red lines in Figure 6. The fitting parameters were denoted as MEAN estimation results. Here, the fitting parameters using *Normal* distribution naturally represented the statistical (MEAN) estimation results. The fitting results and MAP estimation results obtained from indentation Load-1 are listed in Table 2. For the purpose of comparison, the uniaxial experiment data are also listed in Table 2, and the estimation errors with respect to uniaxial experiment data are calculated.

In Table 2, the identified elastoplastic properties from these two MAP points are completely different. The MEAN estimation results showed obvious deviations with respect to these two MAP estimation points and the uniaxial experiment data, and very large estimation errors were observed, e.g., 57.23% for n (MAP point-1 in Table 2). Moreover, the standard deviations (Std. Dev.) of the MEAN estimation values were very large, e.g., the Std. Dev. value was 0.0285 for n. Therefore, the inverse identified elastoplastic parameters under indentation Load-1 were very confused, and this phenomenon should receive due attention.

However, posterior distributions of elastoplastic parameters under indentation Load-2 (1838.24 N) exhibited very convergent and unique results, as shown in Figure 7a–c. The posterior distribution of each parameter shows only one peak value. Moreover, the posterior distribution of elastoplastic parameters was well approximated using the *Normal distribution*. The MAP and MEAN estimation results are listed in Table 3, and they are compared with the tensile experiment values. In Table 3, the identified elastoplastic parameters are very close to the tensile experiment values. The maximum error of the MEAN value was 7.08% (for the estimated n value). In addition, the Std. Dev. values of the MEAN estimation results were relatively small, with respect to the corresponding values obtained from indentation Load-1.

We noted that the posterior result obtained from the proposed measuring method included the probabilistic information of inverse identified elastoplastic parameters, and it was effectively used to interrogate the uniqueness of the inverse problem. Moreover, the result indicated the established method was very effective when the experiment imprint under indentation Load-2 was used in the numerical computation.

**Figure 8:**
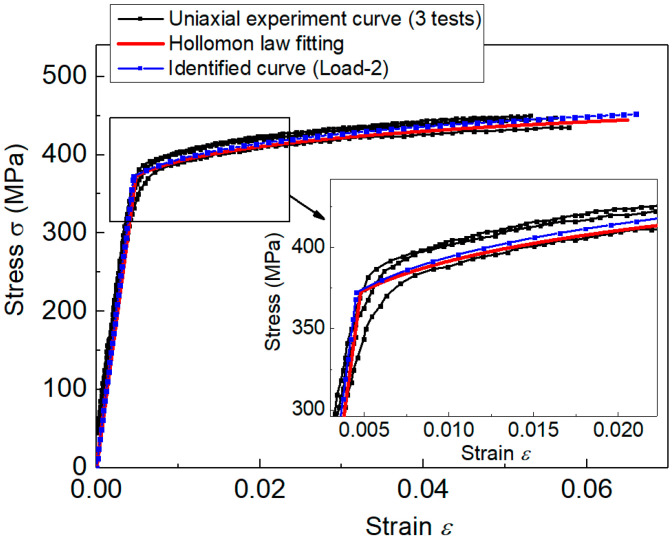
Comparison of the inverse identified stress–strain curve (Load-2) with the uniaxial experiment data.

The MEAN values of Normal distribution essentially represented the statistical estimation results. Thus, they were regarded as the effective elastoplastic parameters. The stress–strain curves represented by the MEAN parameter values from indentation Load-2 are depicted in Figure 8, and they are compared with the uniaxial experiment curves. This showed very good agreement between the tensile experiment curve and the stress–strain curve identified using the established measuring method. Results indicated that the inverse identified stress–strain curve was very accurate and effective.

### 4.2. The Potential Physics Involved in the Non-Unique Posterior of Material Properties Measured by Bayesian Inference Model

**Figure 9:**
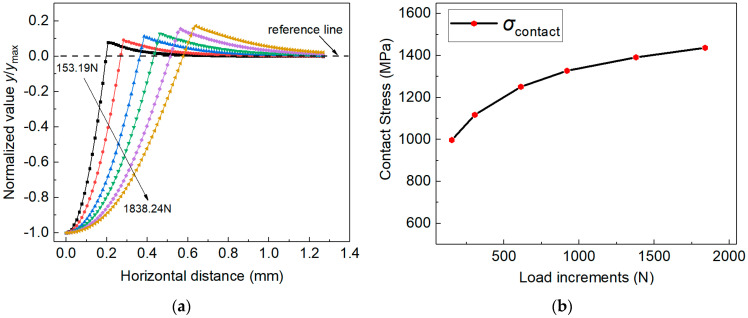
Evolution of indentation responses with the increase in indentation load/depth values: (**a**) the shape of imprint snapshot; (**b**) the contact stress values.

In Section 4.1, the elastoplastic parameters of 2099-T83 Al-Li alloys were identified using the established measuring method and single experiment imprint from two different loads. However, the identified elastoplastic properties were not unique when indentation Load-1 was used, while this non-uniqueness problem was successfully alleviated if the indentation imprint under indentation Load-2 was considered. That is to say, posterior distribution results can be uniquely identified only when a larger indentation load/depth value is used in the established Bayesian inference approach. Here, the potential physics involved in the non-unique posterior estimation results in Figure 6 are further investigated. Figure 9 shows the evolution of the imprint snapshot and contact stress with increases in the indentation load.

Here, the imprint snapshots and contact stress values are obtained from FE simulations using the uniaxial tensile data of 2099-T83 Al-Li alloys. The normalized vertical displacement values are used, and the imprint snapshots obtained from different indentation loads are compared. The contact stress during spherical indentation is approximately calculated using the relations in Equation (16).

(16) $$\sigma_{\mathrm{contact}}=\frac{P_{\max}}{\pi a_{c}^{2}}=\frac{P_{\max}}{\pi \left(2h_{\max}R-h_{\max}^{2}\right)},\;\mathrm{with}\;a_{c}^{2}=\sqrt{2h_{\max}R-h_{\max}^{2}}$$

where $\sigma_{\mathrm{contact}}$ is contact stress, $a_{c}$ is contact radius under indentation, $h_{\max}$ is the maximum loading depth, and R is radius of spherical indenter.

**Figure 10:**
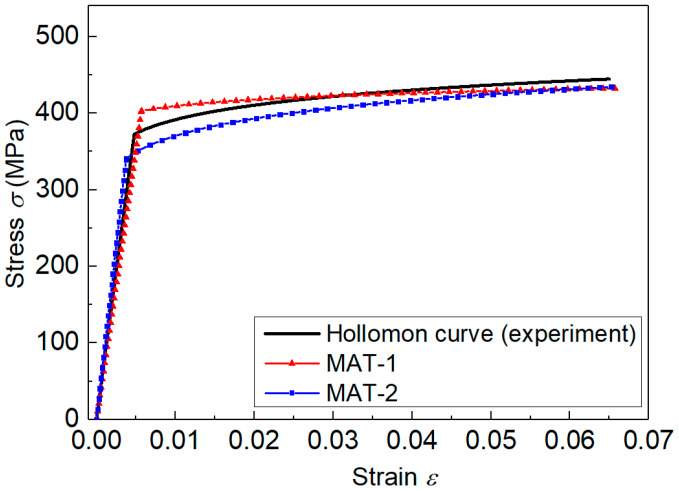
Comparison of stress–strain curves of MAT-1 and MAT-2, with the uniaxial tensile curve.

**Figure 11:**
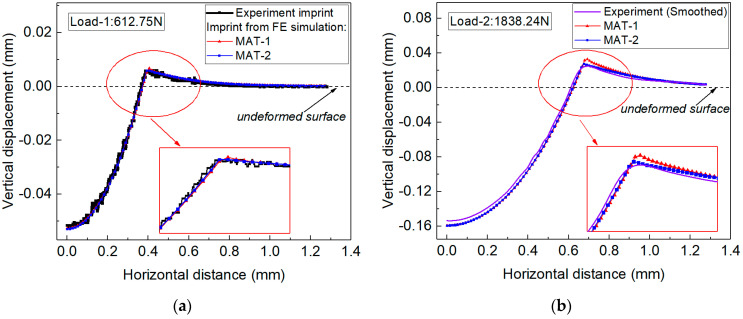
The FE simulated imprint snapshots using the estimated elastoplastic properties of MAT-1 and MAT-2 in situation one: (**a**) simulated imprint snapshots using indentation Load-1; (**b**) simulated imprint snapshots using indentation Load-2.

Figure 9a shows the normalized imprint snapshots, and Figure 9b shows the evolution of contact stress with the load increases. With the increase in load values, the imprint exhibited higher pile-up behavior, and the contact stress was higher. Therefore, it is reasonable that the elastoplastic parameters identified using the residual imprint snapshot under different indentation load were different. The estimated parameters of MAP point-1 and MAP point-2 (in Section 4.1) were denoted as materials MAT-1 and MAT-2, respectively, and their stress–strain curves are compared with the Hollomon curves of Al-Li alloys, as shown in Figure 10. In addition, the estimated elastoplastic parameters of MAT-1 and MAT-2 were used in FE simulations under Load-1 and Load-2, and the corresponding simulated imprint snapshots are also compared with the experiment imprints, as shown in Figure 11a for Load-1, and in Figure 11b for Load-2.

As can be seen from Figure 10 and Figure 11, the stress–strain curves of MAT-1 and MAT-2 are completely different while their corresponding simulated imprint snapshots under indentation Load-1 are very similar (see Figure 11a). Their simulated imprint snapshots were very close to the experiment imprint under indentation Load-1. The above phenomenon in the present work indicated that the different elastoplastic parameters may exhibit indistinguishable imprint snapshots, thus causing the non-unique posterior sampling results. The non-uniqueness of the inverse problem was shown by the posterior marginal distribution using the established measuring method.

We noted that the indentation imprints of MAT-1 and MAT-2 were very different when indentation Load-2 was used in the FE simulation, as shown in Figure 11b. This explained why the inverse identified elastoplastic parameters in situation two became unique when only the imprint snapshot under indentation Load-2 was used in the numerical computation. In the actual experiment, it was hard to know whether the adopted load value was large enough to uniquely identify the material parameters. Therefore, it is suggested to weight the imprints under different load values.

### 4.3. Posterior Distribution of Material Parameters Obtained by Weighing the Imprint Snapshots from Different Indentation Loads

**Figure 12:**
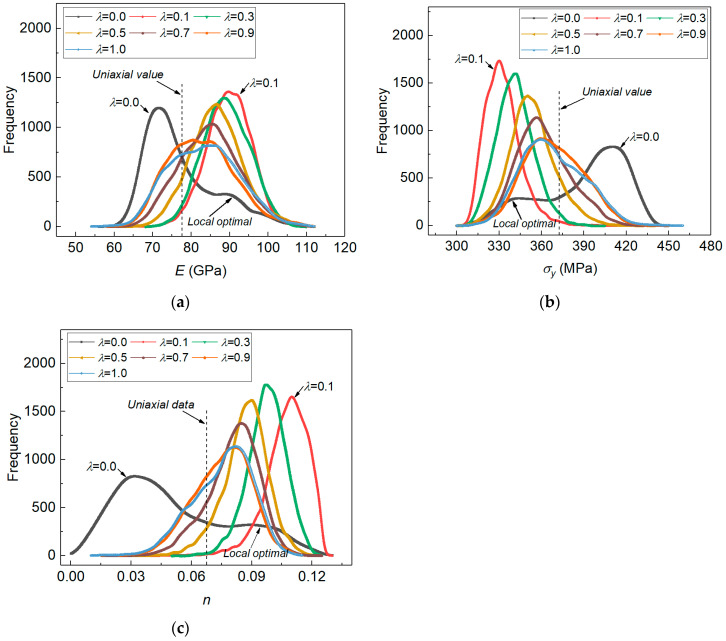
Influence of the weighting coefficient on posterior distribution results of elastoplastic properties, in (**a**) for E; in (**b**) for $\sigma_{y}$; and in (**c**) for n.

In the proposed measuring method, the posterior results were obtained by using only the imprint snapshot under a certain indentation load. The results indicated that the identified elastoplastic parameters may not be unique when the adopted indentation load/depth is not large. In the study, a weighting coefficient was introduced in the measuring method to account for the imprint snapshots obtained from two different indentation loads. Here, the weighing coefficient provided a way to introduce more information on the material deformation characteristics into the numerical computation. Its influence on the estimated results of material parameters will be analyzed. Figure 12 shows the effect of weighting coefficient on the marginal distributions of the identified elastoplastic properties, respectively in (a) for E, in (b) for $\sigma_{y}$ and in (c) for n.

Figure 12 shows that the non-unique posterior results were effectively alleviated when the weighing was applied on the indentation imprints obtained from two different loads. We noted that in the real experiment it was hard to know whether the adopted load was large enough to obtain unique posterior results for the material parameters. Therefore, we used the weighting imprints under two different load values. When the weighting coefficient was within [0.1, 1], the posterior distribution of each material parameter exhibited only one peak value. Moreover, the posterior MAP estimation values were gradually close to the tensile experiment values with weighting coefficient increases. The results indicated that the weighting of imprint snapshots under different indentation loads was able to introduce more information about material deformation characteristics in numerical computation [7,10], and it helped to facilitate uniqueness in the identification of elastoplastic properties using the established method.

**Figure 13:**
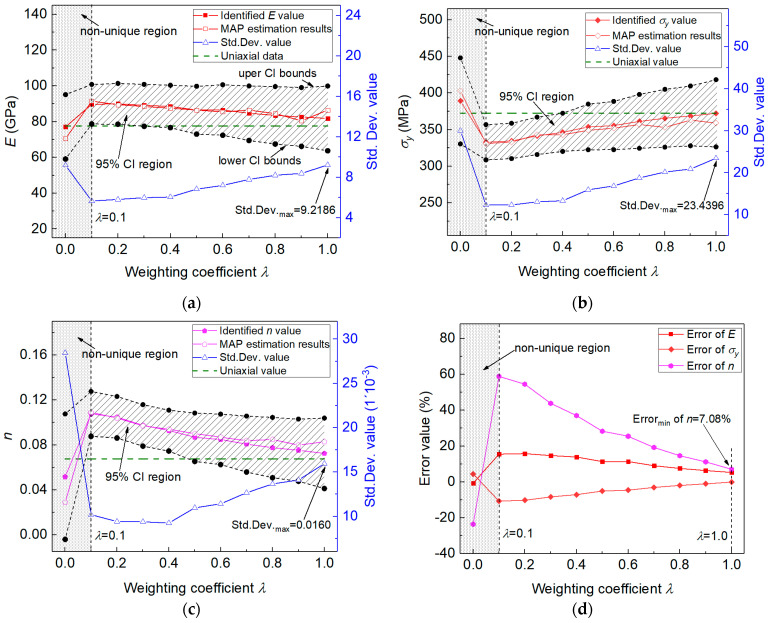
Influence of the weighting coefficient on estimation results of the elastoplastic properties: in (**a**) for E; in (**b**) for $\sigma_{y}$; in (**c**) for n and in (**d**) for the estimation errors.

The influence of the weighing coefficient on the estimated MEAN, MAP values is further shown in Figure 13, in Figure 13a for E, in Figure 13b for $\sigma_{y}$, in Figure 13c for n, and in Figure 13d for the estimation error values. Figure 13 shows that the weighting of indentation imprints under two loads can give unique identification results, e.g., λ∈ [0.1, 1]. When the weighing coefficient was within [0.1, 1.0], the MAP and MEAN estimation results were basically consistent, which indicated the good convergence of the posterior distributions of elastoplastic parameters. The estimated MAP and MEAN values were gradually approximate to the uniaxial data with the increase in weighting coefficient λ.

Based on the *Normal* *distribution* fitting results of the posterior marginal distributions, the confidence intervals (CIs) of the identified elastoplastic properties $\hat{c}$ were determined using the relation: $\hat{c}\in$[$c_{\mathrm{Mean}}-N_{\gamma /2}\cdot c_{\mathrm{Std}.\mathrm{Dev}.}$, $c_{\mathrm{Mean}}+N_{\gamma /2}\cdot c_{\mathrm{Std}.\mathrm{Dev}.}$]. Here, $c_{\mathrm{Mean}}$ and $c_{\mathrm{Std}.\mathrm{Dev}.}$ represent the MEAN and Std.Dev. values from the *Normal distribution* results. $N_{\gamma /2}$ is a constant, and its value is determined by the confidence level (1 − γ), according to the definition of a standard *Normal distribution*. Here, the 95% confidence level was defined, and thus γ was 0.05, and the value of $N_{\gamma /2}$ was 1.96.

The influence of the weighting coefficient on the determined 95% CI of the elastoplastic parameters is also shown in Figure 13. It can be seen from Figure 13 that the 95% CI formed a confidence region, and the estimated MAP and MEAN values were included. As seen in Figure 13, the estimated parameters E and $\sigma_{y}$ exhibited small error values when the weighting coefficient was within [0.1, 1]. Here, the error value of n was relatively large, and it was more sensitive to the variation of weighting coefficient. The main reason for the relatively large error value of parameter n was that the magnitude of uniaxial experiment n value was very small with two decimal places, e.g., uniaxial n value was 0.0678. In the calculation of relative error, uniaxial n value was used as the denominator, thus causing the relatively large error values. That is to say, the estimation error of parameter n was acceptable in the study. Its value was gradually reduced to 7.08% when the weighting coefficient reached 1.0. In addition, in Figure 13, it shows very large Std. Dev. values of the MEAN estimation results in the non-unique region (λ is 0 and only the indentation imprint under Load-1 is used). It seems that relatively small Std. Dev. values were obtained when the weighting coefficient was within 0.1 ≤λ< 1.0. The Std. Dev. value seemed to increase with the increase in the weighting coefficient. Therefore, relatively smaller Std. Dev. values are obtained when the weighting imprints under two different indentation loads are used in numerical computation.

In the study, the proposed numerical method was applied on 2099-Al-Li alloys, and the measuring result obtained from the established measuring method was very effective. This may be ascribed to the high efficiency and accuracy of the POD algorithm used in present work. The POD algorithm served as a very powerful protocol and was able to capture the principal deformation features of the indentation imprint snapshots and provide the optimal representation of an imprint snapshot using the orthogonal basis in well-established sub-space. A Bayesian inverse framework was used in parameter identification, and the potential uncertainties were considered. Posterior sampling results obtained from TMCMC algorithm provided very meaningful probabilistic distribution information on material elastoplastic parameters. Thus, the weighting of imprint snapshots under different indentation loads is suggested. It provides an efficient way to introduce the extra information into the parameter identification process, and thus is very helpful in promoting the uniqueness of the inverse problem. These results represent the progress achieved in the current work in measuring elastoplastic properties by using the experiment imprint from the indentation test. The established numerical method will be applied on other metallic materials, e.g., Al alloys and titanium alloys, and further results will be reported in future work.

## 5. Conclusions

In this paper, an inverse method was established for the measurement of elastoplastic properties of metallic materials by the indentation experiment imprint. The POD algorithm was used to correlate the sub-space coordinates of the indentation imprint with the elastoplastic properties, and the inverse identification was solved using a Bayesian inference approach. The proposed measuring method was applied on 2099-T83 Al-Li alloys. The main conclusions are summarized as following: (1) the potential uncertainties were considered, and the posterior results identified by the new method provide useful probabilistic distribution information of the elastoplastic parameters; (2) posterior distribution of elastoplastic parameters exhibit more than one peak region, when indentation load is not large; (3) weighting of imprint snapshots under different indentation loads can facilitate uniqueness in the identification of elastoplastic parameters using the established measuring method; (4) the identified elastoplastic properties showed good agreement with the tensile experiment, and the established measuring method is very effective.

## Figures and Tables

**Table 1:** Uniaxial mechanical properties obtained from the tensile experiment.

Material	*E* (GPa)	$\sigma_{y}\;(\mathrm{MPa})$	*n*
Al-Li alloys	77.7	372.6	0.0678

**Table 2:** Comparison of the identified MAP, MEAN values of elastoplastic properties using the imprint snapshot under indentation Load-1.

Al-Li Alloys	E (GPa)	$\sigma_{y}\;(\mathrm{MPa})$	n
Uniaxial properties	77.6	372.6	0.0678
Indentation: (Load-1)			
MAP value 1	70.5	403.0	0.029
Error (%)	−9.15	8.16	−57.23
MAP value 2	90.5	341.0	0.085
Error (%)	16.62	−8.48	25.37
MEAN value	77.1	389.3	0.0518
Error (%)	−0.64	4.48	−23.60
Std. Dev.	9.18	30.00	0.0285

**Table 3:** Comparison of the identified MAP, MEAN values of elastoplastic properties using the imprint snapshot under indentation Load-2.

Al-Li Alloys	E (GPa)	$\sigma_{y}\;(\mathrm{MPa})$	n
Uniaxial properties	77.6	372.6	0.0678
Indentation (Load-2)			
MAP value	86.5	359	0.083
Error (%)	11.47	−3.65	22.42
MEAN value	81.8	372.3	0.0726
Std. Dev.	9.2186	23.4396	0.01595
Error (%)	5.41	−0.081	7.08

**Figure A1:**
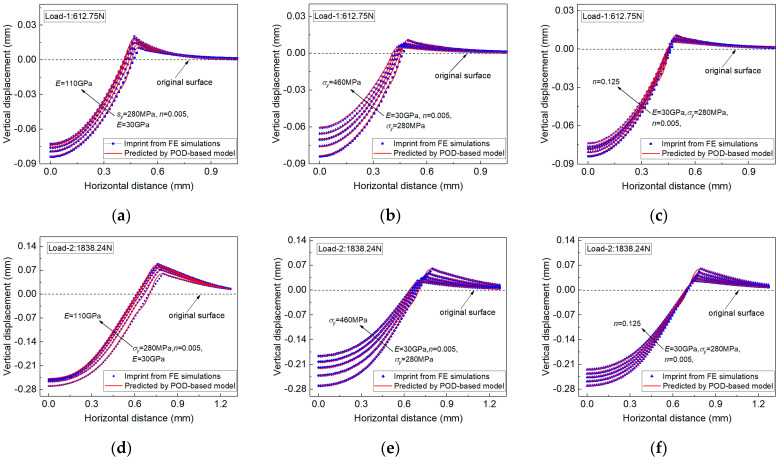
Comparison of the indentation imprints between the FE simulation and those calculated using the parametric relationship in Equation (7): in (**a**–**c**) for indentation Load-1, and in (**d**–**f**) for indentation Load-2.

## Data Availability

The data cannot be shared because it also belongs to an ongoing research.

## Appendix A

The parametric relation between elastoplastic properties c and the sub-space coordinate coefficients $\alpha_{i}$ in Equation (7).

In Equation (7), $\alpha_{i}$ is the coordinate of imprint snapshots $S_{i}$ in sub-space, and each column $\alpha_{i}^{j}$ corresponds to the imprint snapshot $S_{i}^{j}$. Here, subscript i indicates the situation is under the ith indentation load. $\beta_{i}$ is the transposition of matrix $\alpha_{i}$, as $\beta_{i}$ = $\alpha_{i}{}^{T}$. So, the jth row in matrix $\beta_{i}$ is defined as $\beta_{i}^{j}$, and it equals the jth column of matrix $\alpha_{i}$. In the study, extensive simulations were performed to construct the databased $O_{s}$, according to the M combinations in the design space. The 3-order polynomial basis functions can be used to represent the relation between coordinates and elastoplastic parameters in the sub-space, as k = ${\left[1,\;x,\;y,\;z,\;xy,\;xz,\;yz,\;x^{2},\;y^{2},\;z^{2},\;x^{2}y,\;x^{2}z,\;xy^{2},\;y^{2}z,\;xz^{2},\;yz^{2},\;xyz,\;x^{3},\;y^{3},\;z^{3}\right]}^{T}$. Here, the variables x, y and z represent the elastoplastic parameters E, $\sigma_{y}$ and n. The regression coefficient matrix is denoted as $a_{i}$, and each column in $a_{i}$ can be expressed as $a_{i}^{j}$.

Thus, the parametric process obeys the relation: $\beta_{i}^{j}\left(c_{j}\right)$*=*$p^{T}\left(c_{j}\right)\cdot a_{i}^{j}$. Here, the polynomial basis matrix is defined as $K_{b}$, and the jth row in $K_{b}$ is defined as $k_{b}^{j}$, as $k_{b}^{j}$ = $k^{T}\left(c_{j}\right)$. Therefore, the regression process in Equation (7) meets the relationship: $\beta_{i}$ = $K_{b}\left(c\right)\cdot a^{i}$. So, each column $a_{i}^{j}$ in regression matrix $a_{i}$ is obtained by the relation: $a_{i}^{j}\left(c\right)\;$=$\;{\left(K_{b}^{T}K_{b}\right)}^{-}K_{b}^{T}\beta_{i}^{j}$.

Once the correlation between elastoplastic parameters c and sub-space coordinates $\alpha_{i}$ are established in Equation (7), the imprint can be directly calculated using Equation (4). Here, the predicted imprint snapshots are compared with the FE simulation results, and the POD-based model is proven to be effective, as shown in Figure A1a–c for indentation Load-1, and in Figure A1d–f for indentation Load-2. In Figure A1, the shape of indentation imprint is very sensitive to the elastoplastic properties, E, $\sigma_{y}$ and n. Moreover, the indentation imprint predicted by the numerical model is very close to the simulation results. Results indicated the approximation relation in Equation (7) is very reliable.
